# Development and evaluation of a standardised sampling protocol to determine the effect of cleaning in the pig sty

**DOI:** 10.1186/s40813-024-00400-x

**Published:** 2024-10-30

**Authors:** Lisa Dahlin, Ingrid Hansson, Nils Fall, Axel Sannö, Magdalena Jacobson

**Affiliations:** 1 Department of Clinical Sciences, Swedish University of Agricultural Sciences, 750 07 Uppsala, Sweden; 2 Department of Animal Biosciences, Swedish University of Agricultural Sciences, 750 07 Uppsala, Sweden

**Keywords:** ATP-bioluminescence, Hygiene, Residual infectious load, Retrieval rate, Sample-to-sample variability, Test validation, Total aerobic bacteria, Pig pen

## Abstract

**Background:**

All-in, all-out with strict hygienic routines is necessary in modern pig production. Furthermore, a standardised, validated method is needed to quantitatively control the effect of these hygiene protocols. This study aimed to establish a reproducible and reliable sampling method to assess cleaning of the pig pen.

**Methods:**

Sterilised pig faeces were mixed with indicator bacteria (*i.e*. *Enterococcus hirae*, *Escherichia coli*, *Pseudomonas aeruginosa* and *Staphylococcus aureus*) and spread out in a controlled environment. The retrieval rate of three different sampling methods were evaluated; swabbing by (i) a cloth and (ii) a sponge, analysed by standardised bacterial culture and counting of colony-forming units, and (iii) a cotton swab analysed by adenosine triphosphate (ATP) bioluminescence. Two time-points were evaluated during the study; after drying overnight and after manual scraping of the surfaces. To determine sample-to-sample variability, sampling by the cloth and the cotton swab was carried out after manual scraping and further, after high-pressure washing with cold water.

**Results:**

Sampling by the cloth and the sponge showed few differences in in the number of CFU obtained before and after the manual scraping (retrieval rate), whereas the swabs, measuring ATP bioluminescence, showed a very high retrieval rate. Sample-to-sample variability was low for all three methods.

**Conclusions:**

In conclusion, to sample pens for the presence of bacteria, the cloth was assessed as the preferable material, being cheap, easy, specific, and approachable, and with a low sample-to-sample variability. The ATP measurement could have potential for use when evaluating the cleaning of stables, however, threshold values for evaluating the cleaning of a pig sty needs to be developed.

## Background

Healthy pigs are of highest concern in the modern pig production, not only due to farm economics and animal welfare [13, 25], but also to reduce antimicrobial usage and the development of antimicrobial resistance. Prevailing diseases are often caused by opportunistic pathogens that are commonly present in the pig intestine, integument and in the environment [32, 34]. Pigs are generally held in large units with close contact between groups and with high animal density, commonly entailing a high infectious load, that may predispose for outbreaks of endemic diseases [14]. The population structure within pig herds commonly consists of a few older, more resilient, individuals and a majority of younger individuals whose immune system is not yet fully developed [7]. The younger pigs may therefore more easily succumb to disease, with reduction in growth, poor animal welfare, and sometimes even death as a result. To counteract these outbreaks, the practice of all-in, all-out has become the gold standard within the pig industry [1]. By separating animals into units according to age, the risk of older pigs infecting younger pigs may decrease. In-between each batch of animals, the unit is completely emptied, thoroughly cleaned, disinfected, and left to dry, before a new group of animals is installed.

Within human medicine, large emphasis is put on the residual infectious load [27, 37], and within the food safety sector, *e.g*. in slaughterhouses, studies on the prevalence of residual foodborne bacteria have been performed [22]. In the pig pen, few studies have been conducted, and a standardised and reproducible method to measure the infectious load is required. Previously used methods and materials include dip slides, gauze and sponge samplers, and adenosine triphosphate (ATP) measurements [2, 15, 21, 22].

This study aimed to evaluate the retrieval rate of three different sampling methods for the utility in evaluating cleaning protocols in the pig pen. The methods evaluated included a cloth and a sponge, used to analyse the total aerobic count of bacteria, and a swab, used to analyse the presence of ATP bioluminescence to determine the level of cellular material on surfaces. A standardised sampling protocol was developed and evaluated to determine the sample-to-sample variability after mechanical and high-pressure washing removal of faeces.

## Material and methods

### Study location

The study was conducted in the research facilities at the Department of Clinical Sciences, the Swedish University of Agricultural Sciences, Uppsala, Sweden. The stable had been thoroughly cleaned and left empty for approximately one year. The pen floor was a solid concrete floor, covered with an epoxy-free, two-component solvent solution with natural quartz (Piglet Floor®, Flowcrete Sweden AB; Zoric et al. [39]). One week before the start of the study, the stable was cleaned again using a commercial detergent (Grumme Gulsåpa, Orkla, Oslo, Norway) to remove dust, and left to dry.

### Study design

The study was carried out in two parts. In the first part, the objective was to evaluate the retrieval rate of three different sampling methods. Standardised amounts of spiked faeces (10 mL) were applied to 10 × 10 cm areas of the floor and were left to dry overnight before sampling and analyses. Samples that had been contaminated after sampling, *i.e*. during the laboratory work, were discarded and the sampling was repeated on new 10 × 10 cm squares, until ten countable results of each method and occasion was achieved.

**Fig. 1:**
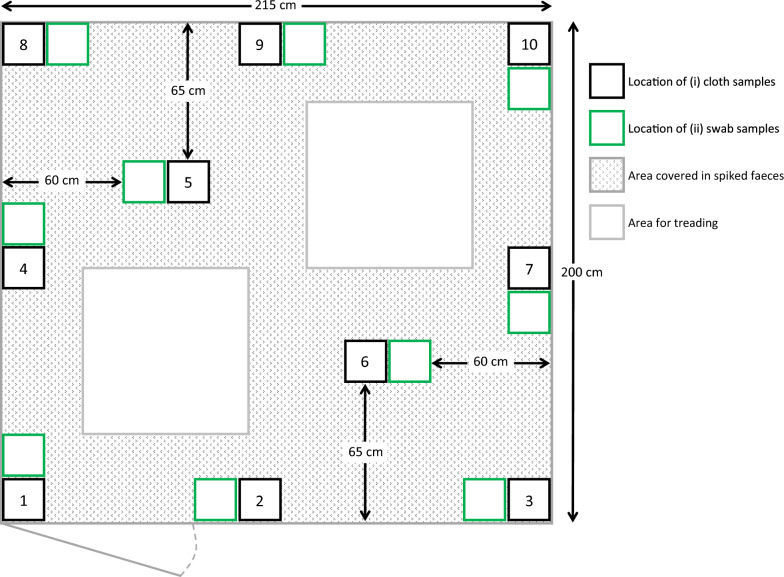
Sampling locations for sample-to-sample variability. Illustration of the slurry distribution, sampling sites and areas for treading. Sampling was performed using two methods; (i) and (iii). The number in each black square indicate the number of each specific sampling location for the cloth (black square) and swabs (adjacent green square)

In the second part of the study, the objective was to determine the sample-to-sample variability using two of the methods and at two different occasions: after manual scraping of the floor and after high-pressure washing of the floor with cold water. Further, critical control points for cleaning in the pen were to be determined. To ensure the same total area and amount of slurry in each pen, the pen floor was measured, marked up, and covered with 10 mL spiked faeces per every 100 cm^2^ of the floor. Sampling was carried out in ten squares of 10 × 10 cm at standardised sites, i.e.; the four corners, four samples adjacent to the middle of each pen wall, and two samples located on each half of the pen, centrally placed (Fig. 1). The entire procedure was repeated three times, each time in a new pen.

Two methods, sampling by (i) a pre-moistened sterile non-woven cloth (Obelis s.a, Brussels, Belgium), and by (ii) a SampleRight™ Sponge Sampler with 10 mL HiCap™ Neutralizing Broth (World Bioproducts LLC, Bothell, Washington, USA), were quantified by bacterial dilution and expressed in colony forming units (CFU). The third method (iii), the 3M™ Clean-Trace™ Surface ATP Test Swab (3M™, St. Paul, Minnesota, USA), was analysed by an adenosine triphosphate (ATP) monitoring device, Clean-Trace™ Luminometer LX25 (3M Health care, St. Paul, USA), expressing the results in Relative Light Units (RLU). The device uses bioluminescence to produce light, and light intensity is directly proportional to the amount of ATP generated in the sample [4]. Since ATP is present in all living cells [6, 29], the RLU reported cannot be interpreted as an exact measure of bacterial presence, instead, it can be perceived as an evaluation of cleaning.

### Faeces preparation

Faecal samples were collected from the specific-pathogen-free herd at the University Research Farm (Lövsta, Uppsala, Sweden). The sterilisation of sow faeces mainly left undigested straw and therefore, approximately 30 L of faeces from nine- to fifteen-week-old grower pigs were instead collected, from 50% of the pen floors in three different units. A three-cm layer of faeces was spread out on a tray and sterilised with steam sterilisation at 121 °C for 16 min, including three vacuum phases and five minutes of drying vacuum (Getinge Gel 6613 ER-2, Getinge AB, Sweden).

To monitor the result of the sterilisation, ten grams of sterilised faeces were placed in a sterile plastic bag, diluted 1/10 with buffered peptone water (BPW; Oxoid CM0509; Basingstoke, UK), and homogenised in a blender for 60 s at 240 rpm (BagMixer® 400 CC, Interscience, Saint Nom la Bretèche, France). From the initial suspension and dilutions 10^–1^ to 10^–2^, 0.1 mL were spread on the surface of 5% bovine blood-agar plates (Swedish Veterinary Agency, Uppsala, Sweden) and incubated at 37 °C ± 1 °C for 24 ± 2 h. The colonies were identified to the species level using Matrix-Assisted Laser Desorption/Ionisation Time-of-Flight Mass Spectrometry (MALDI-TOF MS; Bruker Daltonics, Bremen, Germany). If only spore-forming bacteria such as *Bacillus* spp. were found, the faeces were considered acceptable for use. If any of the indicator bacteria, or more than five colonies of any other bacterial species were present in any dilution, the batch was sterilised a second time. Six out of twenty-one batches required two sterilisations.

### Indicator bacteria and preparation of inocula

The choice of indicator bacteria was based on European and Swedish standard protocols [30, 31], and thus, CCUG17619 *Pseudomonas (P.) aeruginosa*, CCUG15915 *Staphylococcus (S.) aureus* and CCUG46536 *Enterococcus (E.) hirae* were chosen. The recommended CCUG17620 *Escherichia (E.) coli* strain, originally isolated from a human sample, turned out to have a poor survival in the faecal slurry, and instead, a porcine *E. coli* strain was isolated from faeces of healthy grower pigs at the University Research Farm. The strain has been deposited at the Culture Collection at the University of Gothenburg (CCUG77080).

The indicator bacteria were pure-cultured twice on bovine blood-agar and incubated at 37 °C ± 1 °C for 24 ± 2 h, inoculated separately in brain heart infusion broth, and incubated at 37 °C ± 1 °C for 24 ± 2 h. After incubation, quantitative analyses of bacterial broths were carried out through a tenfold serial dilution in 0.1% (v/v) peptone water (Dilucups, LabRobot Products AB, Stenungsund, Sweden). From each of the dilutions 10^–5^ to 10^–8^, 0.1 mL was spread over bovine blood-agar plates and incubated at 37 °C ± 1 °C for 24 ± 2 h. Thereafter, colonies were quantified and the viable count of each bacteria was expressed as log_10_ CFU/mL, the mean concentration of bacteria in the broths being 9.1 ± 0.3 log_10_ CFU/mL.

### Sampling preparations and bacteriological analyses

Before sampling, the cloths were individually placed in sterile plastic bags with 10 mL of BPW and sealed with a plastic clip. The sponge and the swab were pre-prepared in separate packages. All material had room temperature at sampling and clean gloves were used at all times.

The faecal slurry was spiked by thorough mixing of 1 L of sterilised pig faeces with 100 mL of each of the bacterial broths. The mixed slurry measured 0.75 L after mixing, with a mean weight of approximately 780 g. Analysis of viable counts of the slurry was made collecting 10 g of slurry, which was diluted 1/10 with BPW, homogenised and serially diluted. Thereafter, 0.1 mL of the dilutions 10^–2^ to 10^–8^ were analysed, and the viable count of the slurry was expressed as log_10_ CFU/g. In the ATP measurement, the swab was dipped and stirred in 10 mL of the mixed slurry. The mean concentration of the total amount of aerobic bacteria in the slurry was 8.8 ± 0.1 log_10_ CFU/g, and the mean concentration of ATP in 10 mL of the slurry was 3.6 ± 0.05 log_10_ RLU. After spiking, 10 × 10 cm squares outlined by metal frames were covered by 10 mL slurry each, and left to dry overnight. Sampling was carried out by doing ten horizontal and ten vertical strokes back and forth in each square, covering the entire area and using both sides of the cloth and sponge. Using the swab, the entire area was swabbed with both horizontal and vertical strokes, with the swab being rotated at all times. The frames were cleaned and sterilised between each use. Based on the results from the retrieval rate analyses, the cloth and the swab were chosen for further investigations.

In the analyses of the sample-to-sample variability, 4 L of sterilised pig faeces and 400 mL of each of the bacterial broths were mixed, yielding approximately 3.5 L of slurry. To cover a 3.2 m^2^ area of the pen floor, 3.2 L of slurry was evenly distributed and left to dry overnight. The next day, the floor was manually scraped with a plastic pen scraper, sampling was performed in the ten squares as previously described, followed by high-pressure washing with cold water (Kränzle therm 895, Kränzle GmBH & CO. KG, Illertissen, Germany), and a second sampling.

### Cultivation and analysis

Following sampling, 90 mL of BPW was added to the plastic bags with the respective cloths and sponges, the samples were homogenised, and serially diluted. From samples taken after drying overnight, 0.1 mL of the dilutions 10^–2^ to 10^–8^ were spread on the surface of bovine blood-agar plates. The following day, the colonies were counted and the number of bacteria was expressed as CFU/100 cm^2^. The cloth samples collected after scraping were analysed likewise, using 0.1 mL of the dilutions 10^–5^ to 10^–8^, spread over a bovine blood-agar plate, and for the samples taken after high-pressure washing, 0.1 mL of the dilutions 10^–2^ to 10^–6^ was used. The ATP levels were measured within 2 h of sampling by placing the swabs into the ATP monitoring device.

### Statistical analyses

Retrieval rates were calculated using absolute values, expressed in CFU or RLU, comparing the total aerobic bacteria (TAB) or ATP in the spiked slurry before spreading, to the TAB or ATP after drying overnight, and presented as a percentage. To calculate the sample-to-sample variability and evaluate critical control points, bacterial counts (CFU/100 cm^2^) and ATP measurements (RLU/100 cm^2^) were log_10_ transformed. For data management and descriptive statistics, Microsoft® Excel® 2016 (Microsoft Corporation, Redmond, Washington, USA) was used.

## Results

### Retrieval rate

To achieve sixty countable samples, ten per method and occasion, a total of 67 samples were collected, after drying overnight (*n* = 35) and after the manual scraping (*n* = 32). Due to contamination, seven samples were discarded (five cloth and two sponge samples).

Mean RR of the initial amount of TAB was 13.0 ± 4.5% for the cloth, and 4.2 ± 1.5% for the sponge. For the swab, the mean RR of the initial amount of RLU was 44.6 ± 6.7%. The coefficient of variation (CV), which indicates the size of the standard deviation to the mean, for the methods varied largely, from 15.1 to 58.6% (Table 1).

**Table 1:** Mean retrieval rates of the three sampling methods investigated

Sampling method	No. of samples	Mean RR (%)	SD (%)	CV (%)
Cloth	10	13.0	4.5	34.3
Sponge	10	4.2	2.5	58.6
ATP swab	10	44.6	6.7	15.1

Mean retrieval rate (RR), standard deviation (SD) and coefficient of variance (CV) for each sampling method (cloth, sponge and swab), calculated on the absolute number of total aerobic bacteria or relative light units in the spiked slurry before spreading, compared to the corresponding amounts after drying overnight

The mean TAB after drying overnight was 7.9 ± 0.2 log_10_ CFU/100 cm^2^ using the (i) cloth, and 7.4 ± 0.2 log_10_ CFU/100 cm^2^ using the (ii) sponge. After scraping, the mean TAB was 7.7 ± 0.4 log_10_ CFU/100 cm^2^ using the (i) cloth, and 7.5 ± 0.2 log_10_ CFU/100 cm^2^ using the (ii) sponge. By sampling with the (iii) swab, the mean RLU after drying overnight was 3.2 ± 0.06 log_10_ RLU/100 cm^2^, and after scraping; 3.2 ± 0.07 log_10_ RLU/100 cm^2^. The CV for all three methods was low (Table 2).

**Table 2:** Measurement of the total aerobic bacteria (TAB) and relative light units (RLU) after drying overnight and after manual scraping

Sampling occasion	Sampling method	No. of samples	TAB	RLU	CV
Spiked faecal slurry	10 g slurry	6	8.8 ± 0.1 log_10_ CFU/10 g	–	0.01
	ATP swab	6	–	3.6 ± 0.05 log_10_ RLU/10 mL	0.01
After drying overnight	Cloth	10	7.9 ± 0.2 log_10_ CFU/100 cm^2^	–	0.02
	Sponge	10	7.4 ± 0.2 log_10_ CFU/100 cm^2^	–	0.03
	ATP swab	10	–	3.2 ± 0.06 log_10_ RLU/100 cm^2^	0.02
After scraping	Cloth	10	7.7 ± 0.4 log_10_ CFU/100 cm^2^	–	0.05
	Sponge	10	7.5 ± 0.2 log_10_ CFU/100 cm^2^	–	0.03
	ATP swab	10	–	3.2 ± 0.07 log_10_ RLU/100 cm^2^	0.02

Number of samples and log_10_ transformed results of TAB and RLU with standard deviation and coefficient of variation (CV), measured on three different occasions: in the spiked faecal slurry immediately before being spread on the floor, on ten predetermined 10 × 10 cm areas of dried slurry on the floor after drying overnight, and on the same predetermined areas after scraping, using three different methods (the cloth, the sponge and the swab). *ATP*  adenosine triphosphate, *CFU*  colony forming units, *CV*  coefficient of variation

### Sample-to-sample variability and critical control points

**Fig. 2:**
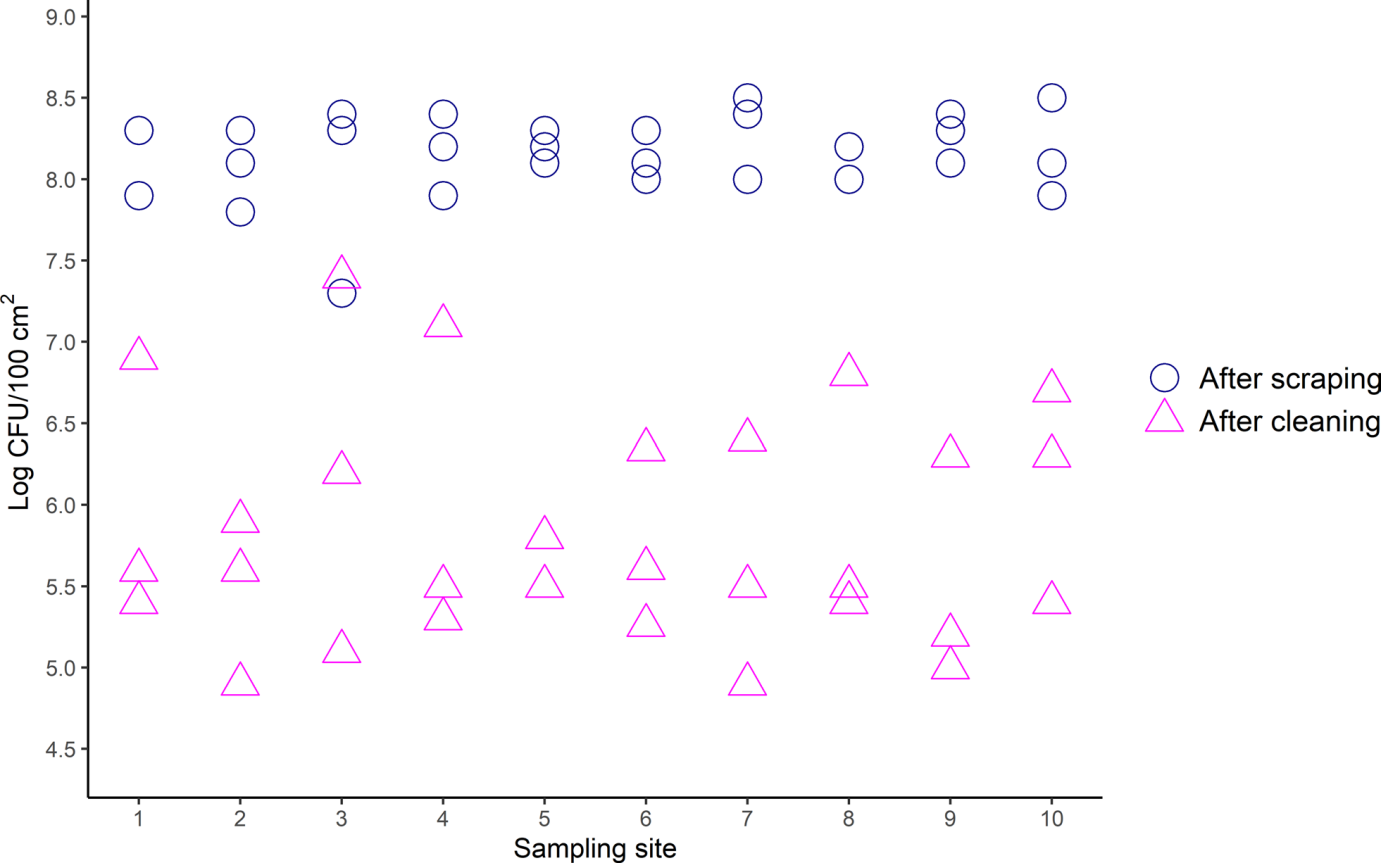
Mean number of TAB for each sampling site, when sampling with a cloth. Mean number of TAB in log_10_ CFU/100 cm^2^, measured on sampling site one to ten on the floor of a pig pen, after scraping (pink triangle) and after high-pressure washing with cold water (blue circle). Each sample site measured 10 × 10 cm. The initial amount of TAB in the slurry was 8.8 ± 0.1 log_10_ CFU

**Fig. 3:**
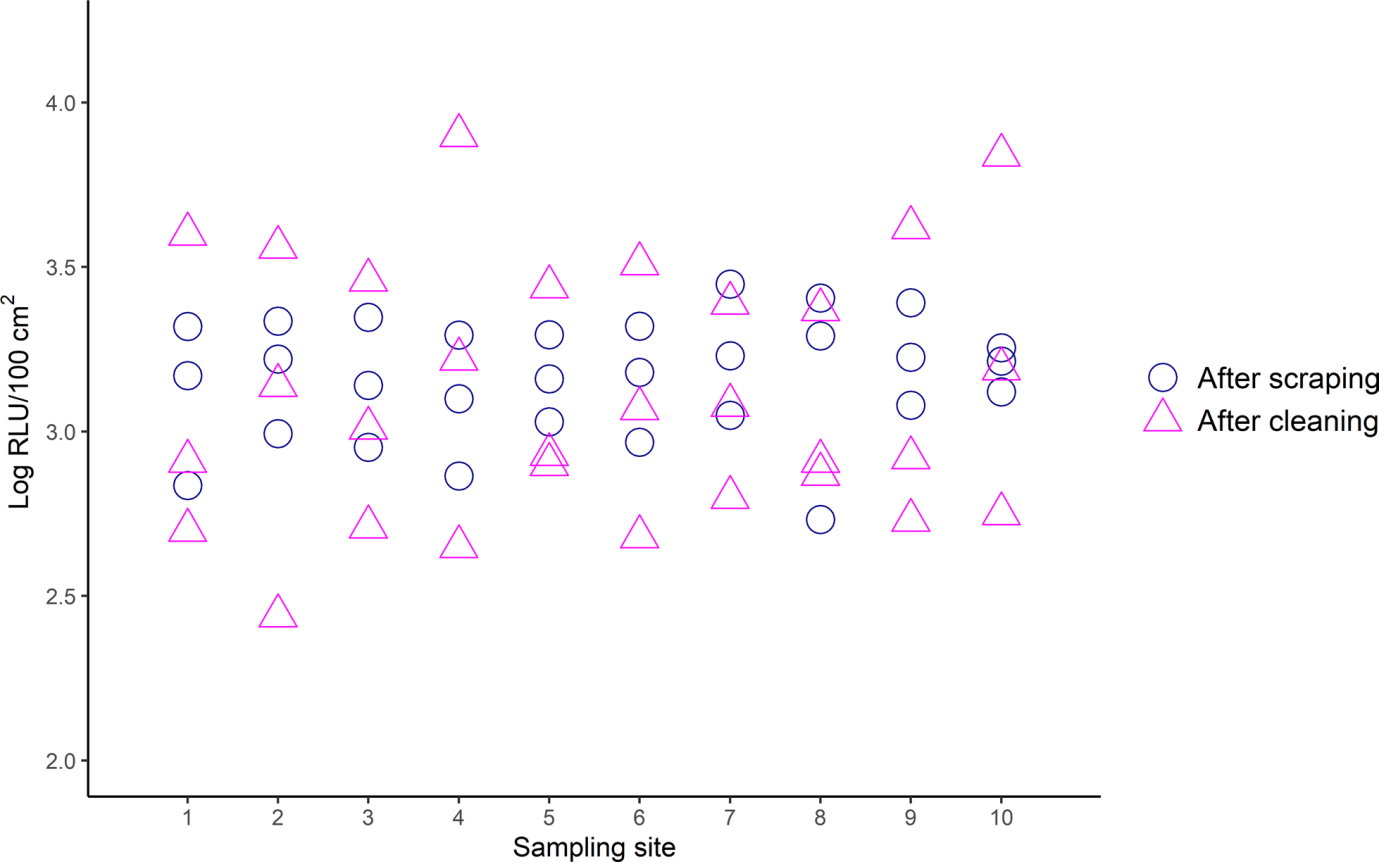
Mean reported RLU for each sampling site, when sampling with a swab. Mean number of RLU in log_10_ RLU/100 cm^2^, measured on the floor of a pig pen on sampling site one to ten, after scraping (pink triangle) and after high-pressure washing with cold water (blue circle). Each sample site measured 10 × 10 cm. The initial amount of RLU in the slurry was 3.6 ± 0.05 log_10_ RLU

In total, 120 samples were collected, 60 samples after manual scraping and 60 after high-pressure washing with cold water, 30 by cloth and 30 by swab at each occasion. No samples were discarded. Following scraping, sampling by cloth resulted in a mean number of viable bacteria of 8.2 ± 0.3 log_10_ CFU/100 cm^2^, and after washing, the mean was 5.8 ± 0.7 log_10_ CFU/100 cm^2^ (Fig. 2). The mean concentration of ATP after scraping was 3.2 ± 0.2 log_10_ RLU/100 cm^2^, and after washing, 3.1 ± 0.4 log_10_ RLU/100 cm^2^ (Fig. 3). The variability between each sampling site was low, and no areas were identified as harder to clean (Figs. 2 and 3).

## Discussion

In the present study, sampling with the cloth rendered a higher retrieval rate, but with a higher standard deviation, than sampling with the sponge, indicating that the cloth is more effective in sampling, but also more unreliable. Comparing the utility of the two methods, the cloth was cheaper, faster and easier to use: the sponge was difficult to lift from its bag, easily got caught on the rough surface and was overall hard to handle without risking contamination. Therefore, the cloth was chosen as the preferred method. In comparison, sampling with the swab resulted in a very high retrieval rate, however, ATP assays measure cell viability based on the occurrence of ATP and does not specifically measure the potential bacterial load, *e.g.* the cultivable aerobic organisms [35], which is of interest regarding animal health and disease prevention [8]. During the sampling for retrieval rate, the measurements for the wet slurry was made by stirring and rotating the swab in 10 mL of the slurry, which could be questioned for comparison with sampling a 10 × 10 cm square. However, sampling was also made directly after spreading 10 mL of wet slurry in 10 × 10 cm squares, where much organic material stuck to the swab, which prevented the Luminometer from reliable readings. The mean RLU measured in the wet slurry on the floor was lower than the control samples, which were taken in clean 10 × 10 cm squares during the same sampling occasion, in the same sampling environment (data not shown). ATP tests are user-friendly and swift, and commonly used within the food industry and human healthcare [22, 23, 26]. However, threshold values for satisfactory cleaning are not established, and would demand a rectification among manufacturers, since reported threshold values varies between brands [26] and sometimes even between devices [24]. Such “pass” grades has been investigated and recommended in several studies, and varies from 0.3 log_10_ RLU/100 cm^2^ to 3 log_10_ RLU/100 cm^2^, depending on the type of facility, surface, and risk level [18]. For the 3M Clean-Trace™ Luminometer used in current study, the manufacturer recommends ≤ 2.4 log_10_ RLU as the “pass” threshold value [3], however, other studies have noted that this threshold value may vary from 2.1 log_10_ RLU/100 cm^2^ [36] to 2.7 log_10_ RLU/100 cm^2^ [11, 23, 29]. Further, the ATP levels in bacteria may differ between species and between individual bacteria, depending on its origin and environmental factors [9, 12, 28].

In any animal setting, a certain occurrence of bacteria is expected and accepted, thus, a higher grade of visually dirty surfaces can sometimes be noted after cleaning, due to *e.g*. contamination from dust falling from the roof. In the sampling with the cloth or sponge, this contamination is probably negligible, on the other hand, the swab might be influenced more due to its low specificity [35]. In the present study, a number of samples taken after cleaning were below the 2.7 log_10_ RLU/100 cm^2^-threshold value established in some hospitals and food processing facilities, and accordingly these areas would pass as clean, however, it is important to still consider the presumptive risk of surviving pathogens. The use of ATP test may not be recommended on soiled areas [4].

Theoretically, the amount of bacteria recovered could have been affected by various factors, such as properties of the floor and the slurry, some bacteria not surviving the desiccation or sticking to the floor despite sampling, and the bacteria’s ability to survive on inanimate surfaces [5, 17, 20]. The sampling for retrieval rate and the subsequent sampling after scraping were made within the same square, and the latter results could therefore have been affected by the first sampling, causing a decrease of matter and bacteria. Further, since the sampling equipment was pre-moistened, some soaking could have taken place, which might have dissolved the dried slurry and eased the next sampling. However, the sampling after drying overnight and after scraping gave similar results, and therefore this effect was considered as negligible.

The sample-to-sample variability using the cloth and swab, respectively, was low. Further, no conclusions could be made regarding the cleaning of specific sampling sites in the pen. Presumably, in a commercial setting, areas that are harder to clean, *e.g*. corners and under feed- and water troughs, would have a higher variability [19, 21, 38]. However, no support for such critical control points could be found in our standardised experimental setting. The experimental design was made to simulate the situation in an average Swedish pig farm, but with a standardised number of a known bacterial flora. The faecal slurry was spread in an even layer, to examine how well a commonly used cleaning procedure will be able to remove some commensal, potentially pathogenic bacteria from different areas (e.g. corners and the slits between floor and wall) in the pig sty. Factors that may affect the initial amount of faeces in various parts of the pen, such as the number of animals, the effect of slatted versus solid floor, the faecal texture and the amount of bedding material, was not included in this study.

The choice of indicator bacteria was based on the European standard protocols SS-EN 14349:2012 and SS-EN 1276:2019 [30, 31]. The four indicator bacteria in SS-EN 1276:2019 were selected for this study, since *Proteus* (*P*.) *vulgaris* used in SS-EN 14349:2012 usually swarms on the agar plates, potentially obstructing the colony counting. Further, *E. coli* was chosen, being of significant clinical relevance [10]. However, the CCUG17620 *E. coli* strain recommended did not grow as expected. In the initial analyses of the wet, spiked slurry only one out of eighty-two colonies were identified as *E. coli*. The spiked slurry was left to dry on the pen floor overnight and sampled again, and only six out of ninety-two colonies were identified as *E. coli* (data not shown). There is a vast diversity within the species *E. coli*, and this particular strain, CCUG17620 (equivalent to ATCC25922) derives from a human clinical sample from 1946, of unknown origin. Thus, this particular strain could have inferior survival and growth in pig faeces. A pig strain was collected and tested; in the wet slurry sample, five out of twenty-four colonies, and in the dried slurry sample taken from the floor, ten out of sixty-three colonies, were identified as *E. coli* (data not shown). Since the pig strain had greater survival, it was chosen for the present study.

In similar studies, dip slides and agar contact plates are commonly used [15, 19, 22], however, these methods were not deemed suitable for the sampling of pen floors, which often are rough and abrasive. The methods also limits the area of sampling [19], and further, in settings with an assumed high bacterial load, there is a risk of plates or slides being unreadable and/or overgrown [16, 19]. In these cases, a bacterial-count method which includes dilution is preferable for correct assessment.

Originally, sow faeces was chosen since farrowing pens was the intended model, however, since the sterilisation left mainly straw, faeces from grower pigs were chosen instead. In the analyses, the presence of spore-forming bacteria was accepted, since these cannot be eliminated completely with sterilisation. However, such samples were discarded if the contamination prevented reliable colony-counting, *i.e*. coalescing with indicator bacteria.

Sampling for sample-to-sample variability was made during the summer months of June and August, which had similar average temperatures (18.1 °C, 16.6 °C), although June had less rainfall than August [33]. All faecal slurry was left to dry over-night, but the slurry spread in August did not visibly dry out as much as the slurry spread in June. However, no significant differences in the results were noted between the different months, hence, the differences in temperature, and potentially in humidity, should not have had a major impact.

The small sample size in this study constituted a limit since isolated values might have influenced the results, and for accurate calculations the most common statistical analyses requires at least thirty values in each group. The CV of the retrieval rates varied largely (15.1–58.6%), indicating a very high variation using different sampling methods, that might have been reduced with a larger sample size. Further, more samples could have resulted in a better and more secure estimation of the true distribution, and if present, potential outliers could have been removed. However, in the study on sample-to-sample variability, the reported values where more alike, with a low CV, which supports these results. Further, bacterial analyses such as dilution series are time-consuming and therefore the number of samples were limited, to ensure that all samples were analysed within the same time-frame, and the number of pig pens that could be used for sampling was also limited.

## Conclusions

In conclusion, to sample pens for the presence of bacteria, the cloth was assessed as the preferable material, being cheap, easy, specific, and approachable, and with a low sample-to-sample variability. The ATP measurement could have potential for use when evaluating the cleaning of stables, however, threshold values for evaluating the cleaning of a pig sty needs to be developed. No critical control points for cleaning could be identified in our experimental setting.

## Data Availability

The datasets used and analysed during the current study are available from the corresponding author upon reasonable request.

## Abbreviations

ATP: Adenosine triphosphate
BPV: Buffered peptone water
CFU: Colony forming units
CV: Coefficient of variation
RLU: Relative light units
RR: Retrieval rate
TAB: Total aerobic bacteria
